# Utilizations of electro-coagulated sludge from wastewater treatment plant *data* as an adsorbent for direct red 28 dye removal

**DOI:** 10.1016/j.dib.2019.104848

**Published:** 2019-11-22

**Authors:** Tadele Assefa Aragaw

**Affiliations:** Faculty of Chemical and Food Engineering, Bahir Dar Institute of Technology, Bahir Dar University, Ethiopia

**Keywords:** EC sludge adsorbent, Adsorption process, Electro-coagulation, Direct red 28 dye, Isotherm, Kinetics, Thermodynamics

## Abstract

This work reported on the adsorptions of direct red 28 dye on to the raw and calcined electro-coagulated, EC, sludge adsorbents collected from the textile wastewater treatment plant. EC sludge adsorbent was prepared with wet treatment by deionized water and calcination at 500 °C. Raw and processed data on main adsorption operation parameters and adsorbent characterization were reported. Also, adsorption isotherm, adsorption kinetics; and thermodynamic models for direct red 28 dye (DR28) removal mechanism on to the raw and calcined EC sludge adsorbents were reported. Instrumental analysis, such as Fourier Transformation infrared spectrometer (FTIR) and Ultraviolet/Visible (UV/Vis) spectroscopy were used for adsorbent characterization and dye absorbance measurement before and after adsorption respectively. UV–Visible spectrometer was used throughout the batch experiment. The effect of adsorption temperature (25 ± 2 °C (ambient), 40 °C, 60 °C and 80 °C), pH (2, 4, 6, 8 and 10), initial dye concentration (20, 40, 60 and 80 mg/L), contact time (10, 20, 40, 60, 80 and 100 min) and adsorbent dosage (0.1, 0.5, 1, 1.5, 2 g/100ml) were examined. For EC sludge adsorbent characteristics, FTIR analysis data is provided as raw and processed data before and after dye adsorption for both raw and calcined EC adsorbent. UV–Vis spectral analysis before and after dye removal with a pH range of 2–10 and batch adsorption experimental data records such as initial dye concentration, solution pH, temperature, adsorbent dosage and mixing time are reported.

**Table:** Specifications Table

Subject	Environmental Science
Specific subject area	Water Science and Technology
Type of data	Table Image Figure
How data were acquired	UV–Visible spectrophotometric data were acquired by generating from the equipment (PerkinElmer lambda 35) with its equipped program. FTIR spectral data were acquired directly from the equipment (JASCO 6600typeA) having equipped program. Zeta potential data was recorded manually using zeta potential Instrument (Malvern Zetasizer Nano ZS series). All batch experimental data were recorded manually as per the procedure described in the methodology section. Percentage removal; adsorption isotherm, kinetics and thermodynamics parameters data were acquired by calculating with their respective model equations using excel. All graphical data were acquired using Origin software version 8.0.
Data format	Raw and Processed
*Experimental Factors*	4 Adsorption temperature (25 ± 2 °C, 40, 60 and 80 °C). 4 Initial dye solution concentration (20, 40,60 and 80 mg/L). 5 Solution pH (2, 4, 6,8 and 10). Using the optimum values of mentioned above, six mixing time (10, 20, 40, 60, 80, and 100 min) and 5 adsorbent dosage (0.1, 0.5, 1, 1.5 and 2g/100ml) with constant 200 rpm were investigated.
*Data Source Location*	Bahir Dar University, Bahir Dar Institute of Technology Bahir Dar, Ethiopia Latitude and longitude; and GPS coordinates 11.5742° N, 37.3614° E and 11°35′37.10″ N 37°23′26.77″ E respectively for Bahir Dar. Sludge from electrocoagulation textile wastewater treatment process was collected from Bahir Dar textile Sharing Company, Bahir Dar
*Data Accessibility*	Repository name: Mendeley Data Data identification number: 10.17632/h2rtgjd6v4.2 Direct URL to data: https://data.mendeley.com/datasets/h2rtgjd6v4/2.

**Table:** 

**Value of the Data** • Investigating azo dye adsorption capacity of sludge from the electrocoagulation effluent treatment process is data promising for scholars and the community. • Batch experimental data are used to decide optimum adsorption capacity of adsorbents from EC sludge and models are used to define the behavior of the adsorption process. • The dataset can be used for industries to implement adsorbents from the EC sludge dye removal and utilization waste sludge as a valuable resource for wastewater treatment and researchers in the university can be used a pre-experiment for other researches. • The dataset can be used for adsorptions of other types of contaminants (heavy metal, anionic nutrients, Phosphate, and nitrate, etc.) from industrial and domestic effluents. • Largely, these data can be used for the removal of different contaminants from the waste stream.

## 1 Data

This work reported on batch adsorption experiments; and adsorption isotherm, kinetics, and thermodynamics models on the adsorbent-adsorbate mechanisms and also EC adsorbent characteristics. Effect of initial dye concentration, the effect of solution pH and effect of temperature in a raw and processed data forms are presented from Mendeley data repository at sheet1 excel file; effect of adsorbent dose and effect of mixing time in a raw and processed data form are presented from Mendeley data repository at sheet 2 excel file. Adsorption isotherm, kinetics; and thermodynamic models experimental values are presented in a raw and processed data form from Mendeley data repository at sheet 3 excel file. UV/Vis spectral analysis before and after adsorption at different pH values of raw and processed data are presented from the Mendeley data repository at sheet 4 excel file. Fourier Transformation Infrared spectral analysis for raw and calcined EC sludge before and after adsorptions of raw and processed data are presented at sheet 5 excel file. Zeta potentials raw and processed data for raw and calcined EC sludge adsorbents at different pH are presented at sheet 6 excel file.

The raw and calcined electro-coagulated adsorbents photographic pictures are presented from Mendeley's data repository as Jpg image attachments. Also, magnetic nature particles from electro-coagulated sludge which is attached to the magnetic bar during the batch experiments are presented from the Mendeley data repository as jpg image attachments.

## 2 Experimental design, materials, and methods

### 2.1 Chemicals and equipment

Hot air oven, jaw crusher, disk mill, sieves, electronic balance, muffle furnace, pH meter, hot plate with a magnetic stirrer, measuring cylinder, test tubes, pipette and what man filter paper centrifuge was used. Powder of dyes concentrated sulphuric acid, sodium hydroxide, potassium bromide, distilled water and were used during adsorbent preparation and each batch experiment.

### 2.2 Adsorbent and adsorbate preparation

#### 2.2.1 Sample collection and physical treatment

Sludge samples were collected from Bahir Dar Textile factory wastewater treatment plant, Bahir Dar, Ethiopia. The sludge was dried at 70 °C using oven, ground and milled to passes less than 200 μm sieve using jaw crusher and disc mill. The desired power was used for wet treatment and calcination but, oversize products have been returned to the crusher. For the separation of impurities from sludge samples in adsorbent preparation such as greases, soluble salts, organic contaminants, wet treatment (beneficiation) process is important and was used [1]. Ground and milled sludge was dispersed in de-ionized water for 24 h and bunged to separate all floatable oils, specks of dust and dissolved solid particles. With decanting the supernatant, the slurry was washed until the unstable particle removed. The suspension after beneficiation has dried at 70 °C to remove absorbed water. Forward after, the beneficiated and dried solids were ground and milled.

#### 2.2.2 Thermal treatment (calcinations)

**Fig. 1:**
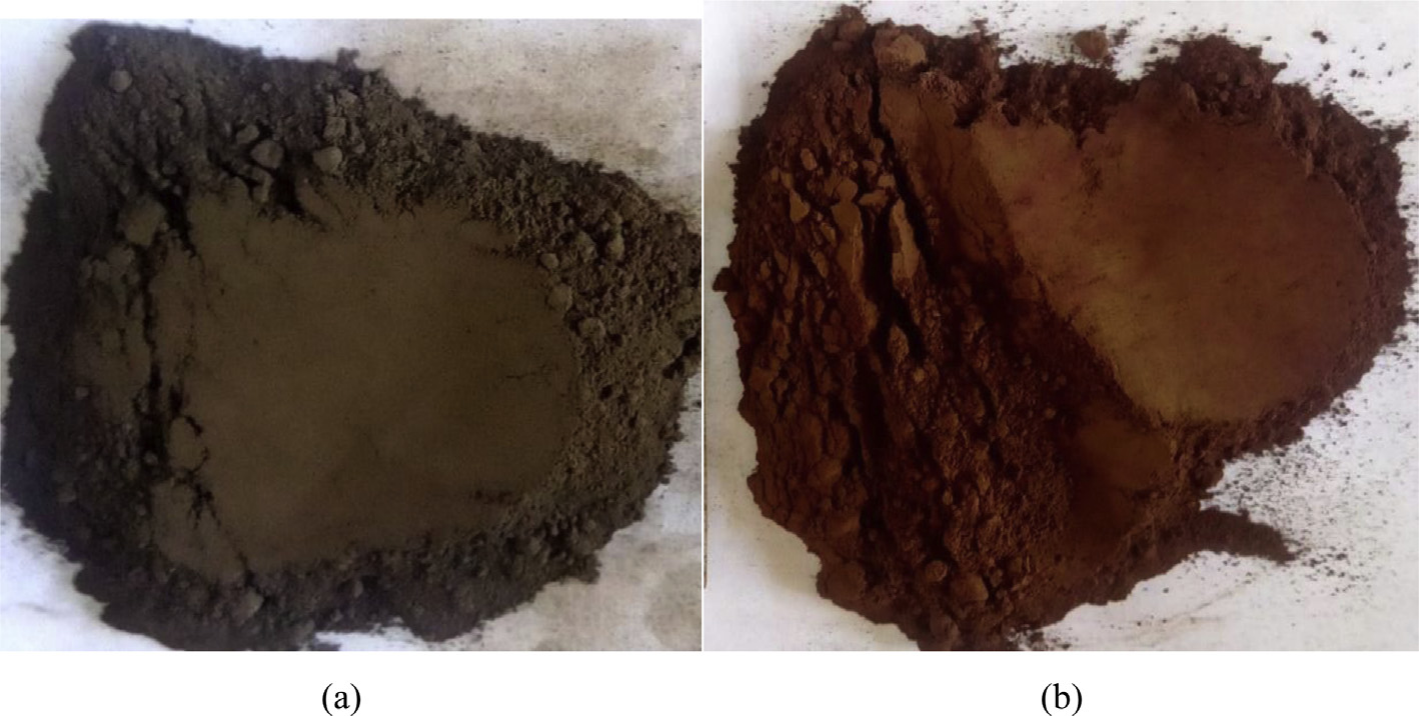
Photographic representations of (a) raw (b) calcined at 500 °C electro-coagulated (EC) sludge.

**Fig. 2:**
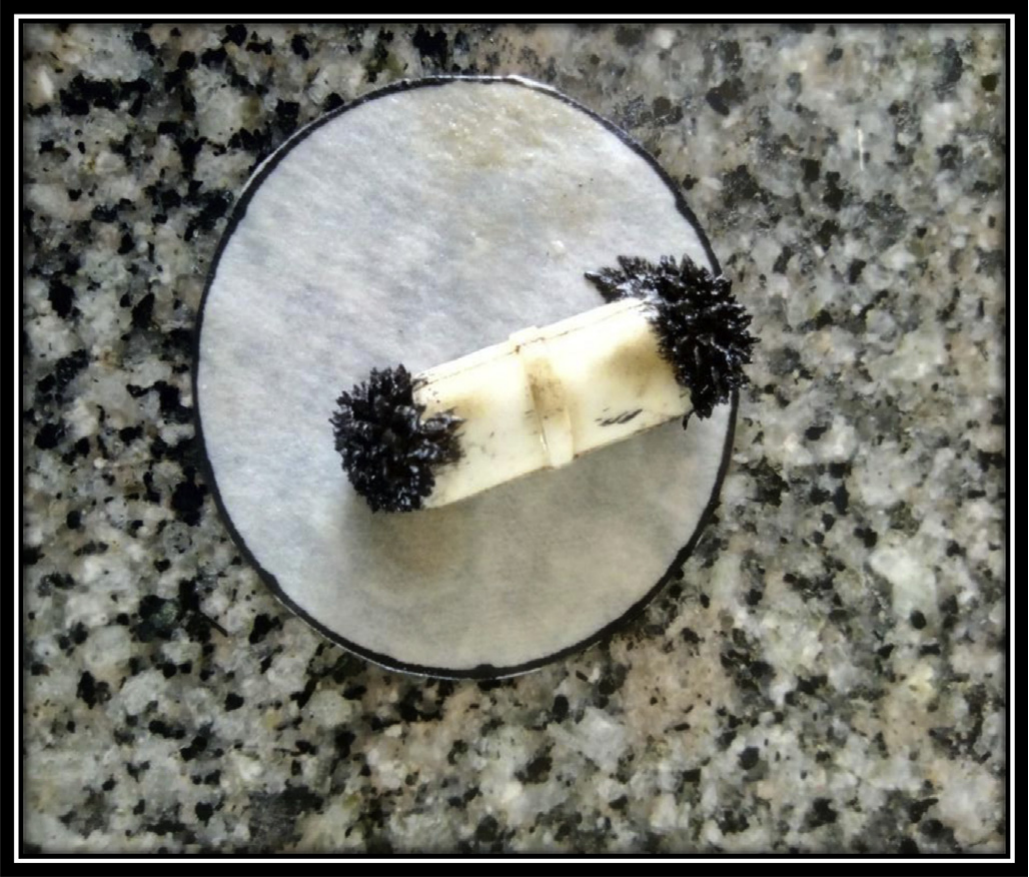
Magnetic nature particles of the electro-coagulated sludge which is attracted to the magnetic bar during the batch experiment.

The wet treated sludge powders were used for thermal treatment at 500 °C for 3 h in a muffled furnace to remove organic carbons and adsorbed water. The calcined EC sludge color changed from grey-black to red. This is due to moisture and volatile organic contaminants from the textile effluents were evolved and oxidative transformation of iron hydroxide to iron oxide is occurred [2] as shown in Fig. 1. Electro-coagulated sludge has magnetic nature due to iron species which is very important to adsorb ionic azo dyes in the solution. This has been confirmed during the batch experiment that the EC adsorbent is attracted to the magnetic bar during a batch experiment as shown in Fig. 2.

#### 2.2.3 Adsorbate preparation

**Fig. 3:**
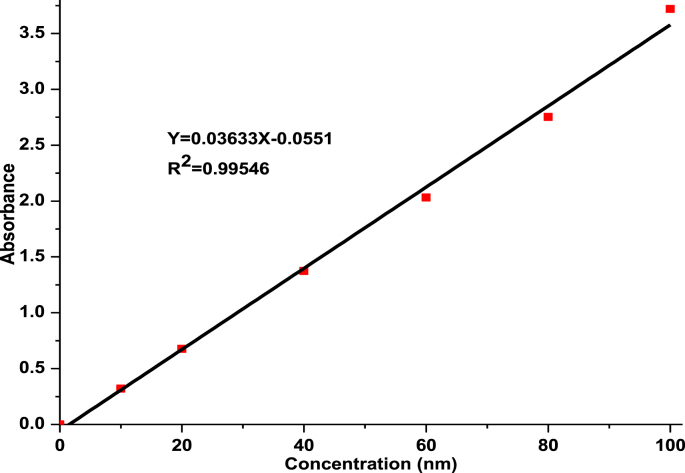
Calibration curve for the standard solutions of Direct Red 28 Dye.

Modal dye, direct red 28 dye, which can be used from the dye industry were taken from commercial marked, Addis Ababa, Ethiopia. Stock solutions were prepared with 0.5 g of powdered dye into 1 L of distilled water making 500 mg/L concentrated solution. Working solutions (20, 40, 60 and 80 mg/L) were prepared with analytical methods. In order to know the maximum wavelength, *λ*_max_, and absorbance of the dye solutions were taken and scanned from 200 to 700 nm using a UV/ViS spectrometer (PerkinElmer Lambda 35) and it was found that 498 nm maximum wavelength. To calculate the final dye concentration from each batch adsorption experiment, a calibration curve was prepared with a standard dye solution of 10, 20,40, 60, 80, 100 mg/L. The linear calibration curve as shown in Fig. 3 was used as a basis for determining the dye concentration variation as a result of the dye adsorption process for each experiment.

### 2.3 Experiment and description

Distilled water was taken in 250 ml of the conical flask for all batch experiments. The predetermined amount of initial dye concentration and EC sludge adsorbent were added and agitated with a magnetic stirrer on a digital hot plate at 200 rpm. Solution pH was adjusted with 1 M solutions of hydrochloric acid and sodium hydroxide before adding the adsorbent. The batch adsorption experiments were performed with dye concentration (20, 40, 60, 80 mg/L), temperature (25 ± 2, 40, 50 and 80 °C) solution pH (2, 4 6, 8, 10), adsorbent dosage (0.1, 0.5, 1, 1.5, and 2 g) and contact time (10, 20, 40, 60, 80 and 100 min). At the end of each experiment, 15 ml of the solutions were withdrawn at a predetermined time and separate the clear supernatant using centrifuge (SIGMA 3-18KS) at 4000 rpm for 10 min. The absorbance after adsorption was measured using UV/Vis spectrometer (PerkinElmer lambda 35) for each run. The final dye concentration was calculated from the calibration curve and the removal efficiency was calculated as with Equation (1) [3]. The equilibrium state concentration (loading) of adsorbate in the solid phase (q_e_, mg/g) and concentration (loading) of adsorbate in the solid phase at any time (q_t_, mg/g) were determined as Equations (2), (3), respectively [4].

(1) $$\text{Removal}\;\text{Efficiency}\left(\text{\%}\right)=\frac{\left({\text{C}}_{\text{o}}-{\text{C}}_{\text{t}}\right)}{{\text{C}}_{\text{o}}}\times 100$$

where $C_{o}$ and $C_{t}$ (mg/L) are the initial and final concentration at time t of the dye in the solution respectively.

(2) $$q_{e}=\frac{V\left(C_{o}-C_{e}\right)}{m}$$

(3) $${\text{q}}_{\text{t}}=\frac{\text{V}\left({\text{C}}_{\text{o}}-{\text{C}}_{\text{t}}\right)}{\text{m}}$$

where q_e_ and q_t_ are the amounts adsorbed (mg/g) at the equilibrium and at any time t respectively. $C_{o}$, $C_{e}$ and $C_{t}$ are the concentration of the dye in the solution (mg/L) at the initial, equilibrium and at any time t respectively; V is the volume of the solution (in Liter), and m is the mass of the adsorbent (in gram).

### 2.4 Adsorbent characterization

FTIR Spectrometer (JASCO 6600typeA) was used to determine the vibration and bending functional groups of raw and calcined sludge's from the electrocoagulation process. 1–100 g sample to KBr ratio was grounded uniformly using mortar and pestle for pellet formation. The pelletized sample with a scanning range of 4000–400 cm^−1^ absorption spectra was recorded. Zeta Potential Instrument (Malvern Zetasizer Nano ZS series) was used at different pH values to determine the surface charge of EC adsorbent. The values were recorded at (acidic media) pH 2, pH 4, pH 6 and (basic media) pH 8 and pH 10.

### 2.5 Analysis of EC adsorbent

**Fig. 4:**
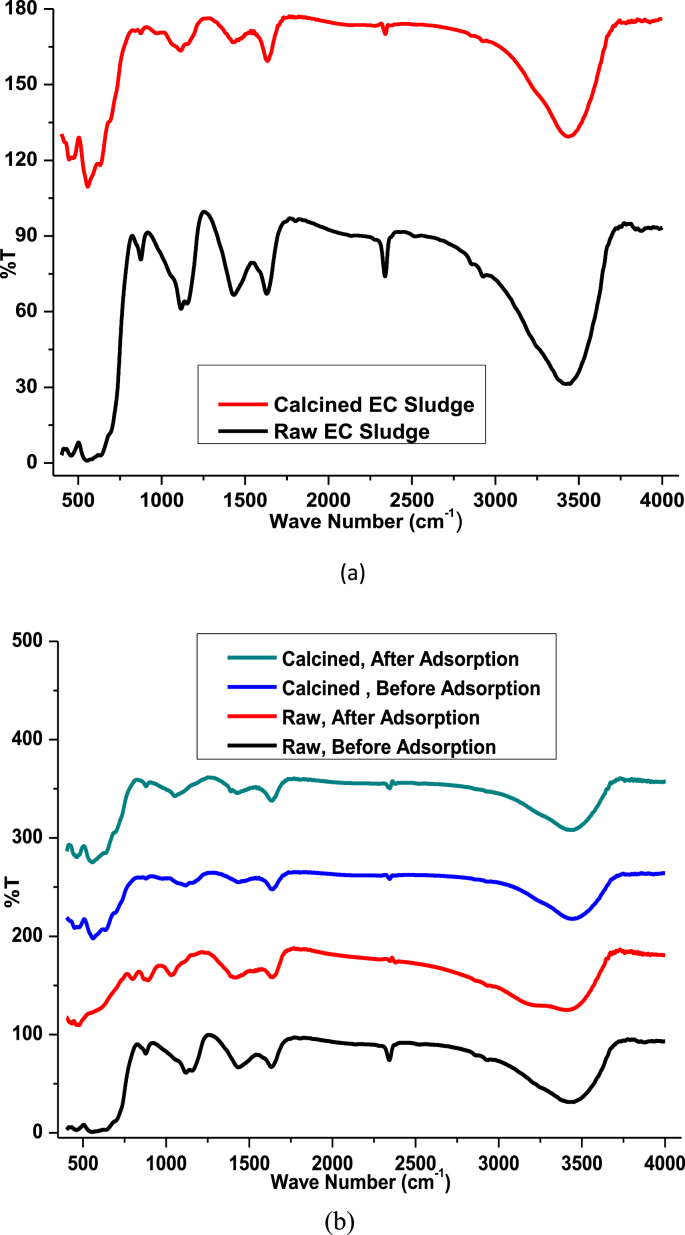
FTIR characterization of (a) raw and calcined EC sludge (b) before and after dye adsorption experiments.

Fourier Transform infrared (FTIR) spectra were used to determine vibrational and bending spectra of raw and calcined electro-coagulated sludge in the range of 4000–400 cm^−1^ [5] by using a JASCO (6600typeA) Frontier Spectrometer as depicted from processed data in Fig. 4 a. The band appears at 1097 cm^−1^ is typically metal hydroxyl (M − OH) stretching vibration [6]. Also before and after adsorptions of dye both in raw and calcined EC adsorbent were analyzed to determine percentage transmittance and to identify new functional group spectral peaks if any as shown processed data in Fig. 4 (b).

**Fig. 5:**
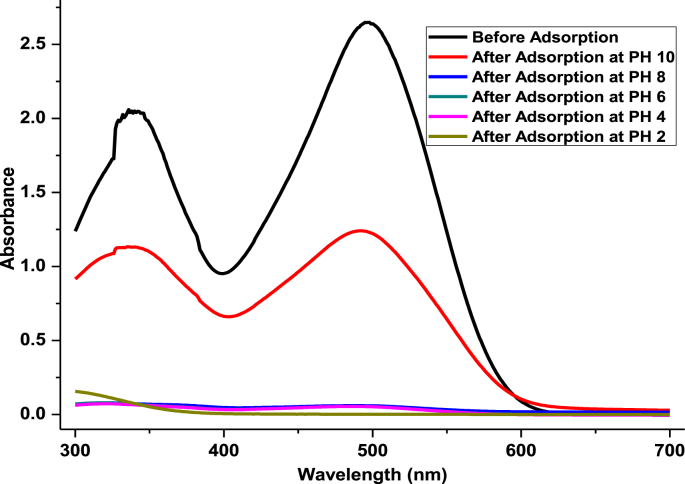
UV–Vis spectra of before and after direct red 28 dye adsorption on to calcined EC adsorbent at different pH solutions.

UV/Vis spectral analysis is very important before and after treatments of the dye solution to determine the intensity of peaks at a specified wavelength whether the peak intensity is disappeared or not. Thus, before and after dye adsorption spectra were recorded in a range of 300–700 nm wavelength at different pH solutions. The processed graphical representation is presented in Fig. 5. Absorption peaks at 350nm and 498 nm were disappeared at the pH of 2, 4, 6 and 8. But, at the far basic media at pH 10 the peak is similar to before adsorption of dye solutions.

### 2.6 Isotherm, kinetics and thermodynamics study

#### 2.6.1 Adsorption isotherm model

The raw and processed data of adsorption isotherm is presented in Mendeley Data repository of Microsoft Excel sheet 3. Langmuir and Freundlich isotherm models were used to determine the relationship between dye ions from the solution adsorbed on EC adsorbents and remaining in the solution. The Langmuir isotherm model determines that adsorption occurs in certain places and within the adsorbent. In addition, the sorption of dyes occurs within homogeneous monolayers without any interactions between dye solutions at the adsorbent surface. But, the Freundlich adsorption isotherm model can be used that the adsorption of dye ions from the solution occurs at heterogeneous, varied mixture, surfaces and active sites with different energies. The Langmuir and Freundlich model linear Equations can be governed as Equations (4), (5) respectively [7,8].

(4) $$\frac{{\text{C}}_{\text{e}}}{{\text{q}}_{\text{e}}}=\frac{{\text{C}}_{\text{e}}}{{\text{q}}_{\text{m}}}+\frac{1}{{\text{q}}_{\text{m}}{\text{K}}_{\text{L}}}$$

(5) $${\text{logq}}_{\text{e}}=logkf+\frac{1}{\text{n}}{\text{logC}}_{\text{e}}$$

where q_m_ is sorption capacity (mg/g), K_L_ is sorption energy (L/g), K_f_ and n are the Freundlich model constants and indicating the relationship between sorption capacity and sorption intensity, respectively. If n = 1, n > 1, and n < 1, then the sorption process would be the linear, physical, or chemical in its nature respectively.

The calculated Langmuir and Freundlich adsorption isotherm constant parameters presented in Table 1.

**Table 1:** List of calculated parameters of the Langmuir and the Freundlich adsorption isotherm models for the adsorption of direct red 28 dye on to raw and calcined EC adsorbent.

Types of EC adsorbents	Langmuir Isotherm Parameters			Freundlich Isotherm Parameters		
	q_m_ (mg/g)	K_L_ (L/mg)	R^2^	K_f_ (mg/g)	n	R^2^
Calcined	1.252	17.75	0.9186	0.905	5.847	0.7156
Raw	1.262	14.40	0.9504	1.114	1.275	0.9844

#### 2.6.2 Adsorption kinetic model

The raw, calculated and processed data of kinetics are present in Mendeley Data repository from Microsoft Excel sheet 3. The kinetic model was used to determine reaction kinetics of adsorption mechanism as a dependency of mixing time. The raw and processed data of the effect of mixing time on the adsorption process is presented from Mendeley Data repository of Microsoft Excel sheet 3 (Table 2).

**Table 2:** List of calculated parameters for pseudo-first and second-order Adsorption Kinetic of direct red 28 dye on to raw and calcined EC adsorbent.

Types of adsorbents	q_e_, exp. (mg/g)	Pseudo First Order Parameters			Pseudo Second Order Parameters		
		q_e_ (mg/g)	K_1_ (min^−1^)	R^2^	q_e_ (mg/g)	K_2_ (g/mg*min)	R^2^
Calcined	1.942	0.044	0.048	−0.13511	1.930	−4.134	0.99996
Raw	1.937	0.019	0.029	−0.18265	1.926	−4.147	0.99996

The linear Equations for pseudo-first and second-order models are presented in Equations (6), (7) respectively.

(6) $$log\left(qe-q_{t}\right)=logq_{e}-\frac{k_{1}}{2.303}t$$

(7) $$\frac{t}{q_{t}}=\frac{1}{k_{2}{q_{e}}^{2}}+\frac{1}{q_{e}}t$$

#### 2.6.3 Thermodynamic behavior

The raw and processed data of adsorption thermodynamics is presented in Mendeley Data repository of Microsoft Excel sheet 3. The thermodynamic property can be used for the determinations of exothermic and endothermic temperature dependency reactions in terms of Gibb's free energy, enthalpy, and entropy. The values of standard change Gibbs free energy, enthalpy, and entropy is obtained from Equations (8), (9) [7]. In thermodynamics, the exothermic nature of the adsorption process tells us the increase of the atomic or molecular species release from solid surface and re-enters the liquid phase [9,10].

(8) $$\text{Δ}G^{0}=\text{Δ}H^{0}-T\text{Δ}S^{0}$$

(9) lnKc=−ΔG0RT=ΔS0R−ΔH0RT

where ΔG^0^ = Standard change free Gibbs energy (kJ mol^−1^), $\Delta {\text{H}}^{0}$ = Standard change enthalpy (J mol^−1^), $\Delta {\text{S}}^{0}$ = Standard change entropy (J. mol^−1^K^−1^) and R = Universal gas constant (8.314 J mol^−1^K^−1^). K_c_, the equilibrium constant. K_c_ is the ratio of the equilibrium concentration of the dye (q_e_) adsorbed to adsorbent compared to the Van't Hoff equation as equilibrium dye concentration in solution (C_e_). The calculated values of standard change Gibbs free energy, enthalpy, and entropy constant parameters are presented in Table 3.

**Table 3:** Thermodynamic parameters for the adsorption of direct red 28 dye on to raw and calcined EC adsorbent.

Types of adsorbents	$\text{Δ}G^{0}$ [KJ.Kmol^−1^]				$\text{Δ}H^{0}$[KJ.Kmol^−1^]	$\text{Δ}S^{0}$[KJ.Kmol^−1^K^−1^]
	**Temperature [K]**					
	298.15	313.15	333.15	353.15		
Calcined	−4.482		−6.027	−7.572	19.712	0.077
Raw	−4.246		−5.786	−7.326	19.862	0.076
